# The Role of Angiotensin II in Glomerular Volume Dynamics and Podocyte Calcium Handling

**DOI:** 10.1038/s41598-017-00406-2

**Published:** 2017-03-22

**Authors:** Daria V. Ilatovskaya, Oleg Palygin, Vladislav Levchenko, Bradley T. Endres, Alexander Staruschenko

**Affiliations:** Department of Physiology, Medical College of Wisconsin, Milwaukee, Wisconsin 53226 USA

## Abstract

Podocytes are becoming a primary focus of research efforts due to their association with progressive glomeruli damage in disease states. Loss of podocytes can occur as a result of excessive intracellular calcium influx, and we have previously shown that angiotensin II (Ang II) via canonical transient receptor potential 6 (TRPC6) channels caused increased intracellular Ca^2+^ flux in podocytes. We showed here with patch-clamp electrophysiology that Ang II activates TRPC channels; then using confocal calcium imaging we demonstrated that Ang II–dependent stimulation of Ca^2+^ influx in the podocytes is precluded by blocking either AT_1_ or AT_2_ receptors (ATRs). Application of Ang(1–7) had no effect on intracellular calcium. Ang II-induced calcium flux was decreased upon inhibition of TRPC channels with SAR7334, SKF 96365, clemizole hydrochloride and La^3+^, but not ML204. Using a novel 3D whole-glomerulus imaging *ex vivo* assay, we revealed the involvement of both ATRs in controlling glomerular permeability; additionally, using specific inhibitors and activators of TRPC6, we showed that these channels are implicated in the regulation of glomerular volume dynamics. Therefore, we provide evidence demonstrating the critical role of Ang II/TRPC6 axis in the control of glomeruli function, which is likely important for the development of glomerular diseases.

## Introduction

The understanding of molecular mechanisms leading to protein shedding in the urine is key for developing targeted treatment for glomerular diseases. Progressive chronic nephropathies are typically characterized with podocyte depletion and associated glomerulosclerosis^1^. As a result of podocyte loss occurring in response to various pathological stimuli, glomerular filtration barrier (GFB) gets damaged, which initiates the onset of proteinuria. One of the key findings of the past decade was a discovery of a gain-of-function mutation in the podocytic transient receptor potential canonical channel 6 (TRPC6), which was found to cause Focal Segmental Glomerulosclerosis (FSGS)^2–4^. This genetic breakthrough stimulated the studies of TRPC6 as a key mediator of Ca^2+^ flux in the podocytes^5^, and aberrant Ca^2+^ handling by the podocyte is now considered an important determinant of its injury and loss^6–10^.

Persistent elevation in proteinuria is a sign of declining kidney function, which can result from various pathological stimuli^11–13^. According to National Institute of Diabetes and Digestive and Kidney Diseases, diabetes and hypertension are the leading cause of end-stage renal disease (ESRD). Both landmark genetic research^14^ as well as renal patients biopsies studies^15^ recognize the glomeruli podocytes damage as a main cause of albuminuria. Podocytes are the critical elements of the GFB, and as they have limited proliferative capacity, their ability to counter stress plays a crucial role in the development of proteinuric glomerular diseases^16, 17^.

There is a number of physiological stimuli which can lead to glomeruli damage and proteinuria, and among these Angiotensin II (Ang II)^18–21^ is one of the major ones; it was demonstrated that interstitial levels of Ang II are increased in patients with progressive glomerulopathies^4^, and it mediates chronic renal inflammation and fibrosis^22^. Further studies revealed that enhanced AT_1_ receptor signaling in podocytes leads to proteinuria and FSGS^23^, and inhibition of AT receptors is effective against proteinuria^24, 25^.

Numerous research efforts were devoted to unraveling the connection between Ang II and TRPC channels^18, 26–28^. There is still controversy about which TRPC channel, TRPC5 or TRPC6, is responsible for the enhanced calcium signaling in the podocyte^10, 20, 29, 30^. In our recent studies^21, 31^ we have shown that TRPC6 is the main channel activated by Ang II in podocytes and that this pathway is implicated in podocyte injury occurring in diabetic nephropathy.

Podocyte, however, should not be studied in isolation from the rest of the glomeruli filtration apparatus. Under the influence of the circulating factors glomeruli function changes as a whole, resulting in altered filtration rate. It has been long known that glomerular contractions in response to Ang II correlate with glomerular function^32, 33^, but the molecular mechanism of this complex process is not entirely known. Savin *et al*. demonstrated in cultured glomerular epithelial cells^34, 35^ that both AT_1_ and AT_2_ receptors of Ang II are involved in regulation of intracellular calcium. However, in order to fully understand the relation between changes in Ang II levels and GFR, a more comprehensive approach should be applied, where glomerulus is studied as an entity that integratively responds to Ang II involving podocytes, mesangium, and vasculature. The current study was aimed at delineating the involvement of TRPC-mediated calcium influx in Ang II-AT_1_/AT_2_ receptors signaling pathway, and testing the hypothesis that this signaling axis could be implicated in the glomeruli volume dynamics, which is currently viewed to be cooperatively mediated by all types of glomerular cells^36^.

## Results

### AT_1_ and AT_2_ receptors both mediate the volume response of glomeruli to an oncotic gradient of albumin

**Figure 1:**
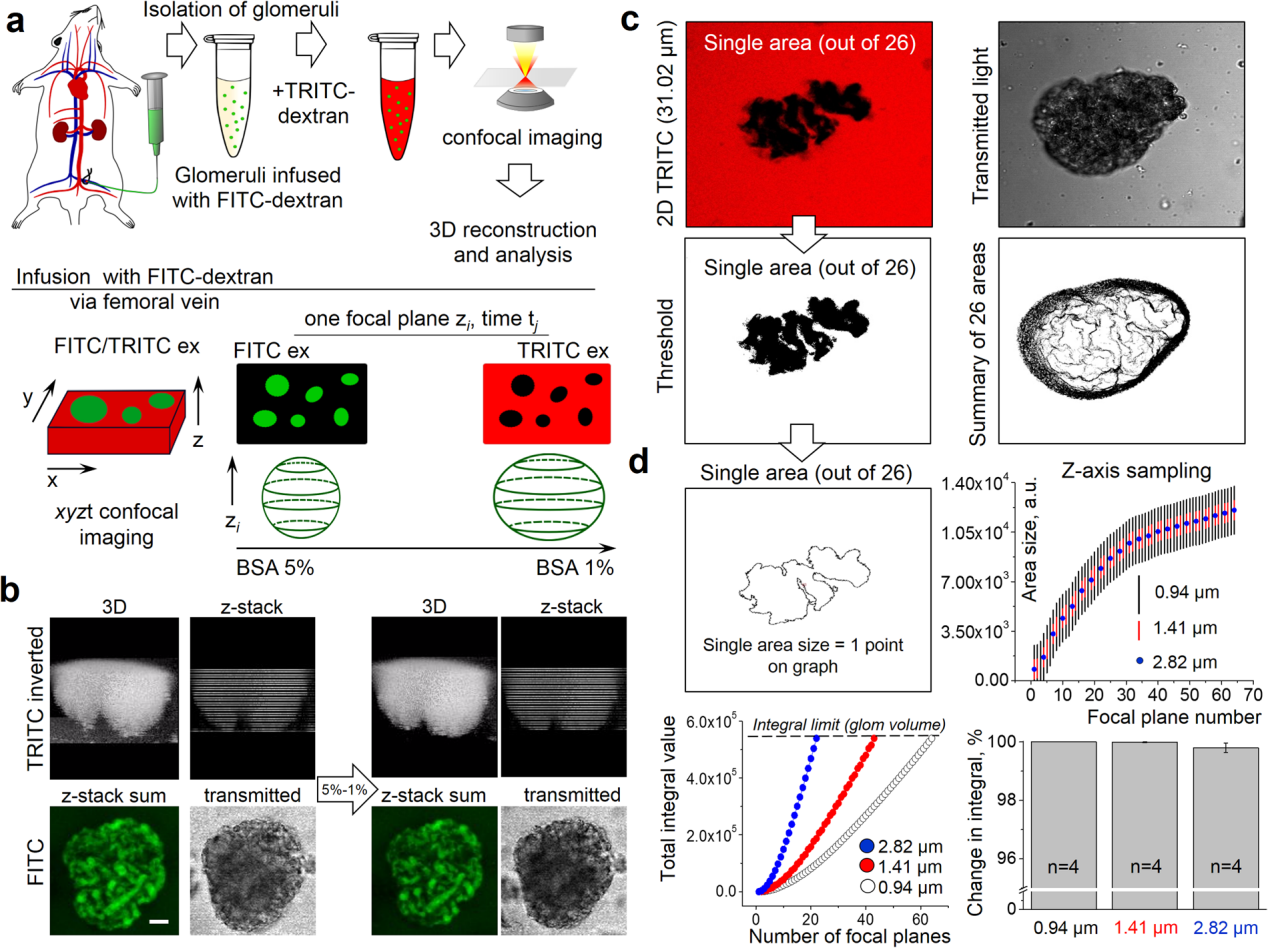
Schematic explanation of the measurements of glomeruli permeability and volume changes. (**a**) General steps of the glomeruli permeability assay: surgical preparation of the anesthetized animal and injection of 150 kDa FITC-dextran into circulation via femoral vein; glomeruli isolation, incubation and addition of 150 kDa TRITC-dextran into the solution containing FITC-labeled glomeruli; fast xyzt confocal fluorescence microscopy scanning during the changes in oncotic gradient (BSA 5% to 1%); 3D reconstruction and image analysis of glomerular volume and inner fluorescence changes. (**b**) Example of glomerular volume reconstitution and image analysis for the permeability assay. Fluorescence intensity was summarized from corresponding FITC values and subsequently analyzed. Also shown is a transmitted light image of a single glomerulus. Arrow illustrates solution change from 5% to 1% BSA. Scale bar is 20 µm. (**c**) Glomeruli volume reconstitution. To assess the glomerulus volume, images from 26 focal planes with a slice thickness of 2.82 μm were obtained along the z-axis of each glomerulus. Shown here (“2D TRITC”, left upper panel) is an example of a scanned area from the middle of the imaging stack (slice 11 out of 26, which corresponds to 31.02 μm on z-axis), that reveals a detailed structure of glomerulus (black) covered with TRITC-dextran (red). Adjusted threshold level used to calculate slice area in the Analyze Particles Module (ImageJ), as well as a 3D summary of all areas for current glomerulus are shown in the right panel. (**d**) Glomerular volume calculation. Shown on the left (upper panel on *D*) is a single area used to calculate the glomerulus volume. Right upper panel demonstrates three different sampling rates (2.82 (26 z-slices), 1.41 (52 slices) or 0.94 μm (78 slices)), each point on the graph corresponds to the size of the single area (shown on left). Total integral value for each sampling rate, and the summary bar graph (lower panel) demonstrate that independently of the size of the z-axis step, total integral value (glomerulus volume) riches the same level.

The instrumental work by Höhne *et al*.^37^ suggested that the integrity of the filtration barrier could be undermined by changes in podocyte dynamics, which leads to increased albumin permeability, and is being reflected in the observed oscillatory glomerular contractions. Following this lead, we modified the classic method suggested by Savin *et al*.^38^, which was designed to measure volume response of glomeruli to an oncotic gradient generated by defined concentrations of albumin. Figure 1a illustrates the schematic of the modified method, featuring the combination of fluorescent dextrans and fast confocal imaging of the glomerular volume, which allows precise characterization of permeability changes in response to various drugs. Change of the medium (in presence or absence of the tested compounds) from 5% to 1% BSA produces an oncotic gradient across the glomerular capillary wall and results in a change in glomerular volume and brightness (targeted by TRITC and FITC fluorescence, respectively), which correlates with glomerular permeability to protein.

Examples of glomerular volume reconstitution and fluorescence dilution analysis for the permeability assay are shown on Fig. 1b. For the calculation of glomerular volume 26 areas from consecutive focal planes of TRITC fluorescence were summarized for every data point. Changes in fluorescence intensity were summarized from corresponding FITC values. To assess the glomerulus volume images were taken every 2.82 μm (Fig. 1b,c) for following analysis. Shown on Fig. 1c (“2D TRITC”, left upper panel) is an example of a scanned area from the middle of the imaging stack (slice 11 out of 26, which corresponds to 31.02 μm on z-axis), that reveals a detailed structure of glomerulus (black) covered with TRITC-dextran (red). We also analyzed volume of glomeruli when the number of focal planes (z-stack sampling rate) was increased. As shown on Fig. 1d changes in z-axis step size (tested sampling rates were 2.82, 1.41 and 0.94 µm, respectively) did not affect calculation of glomerular volume.

**Figure 2:**
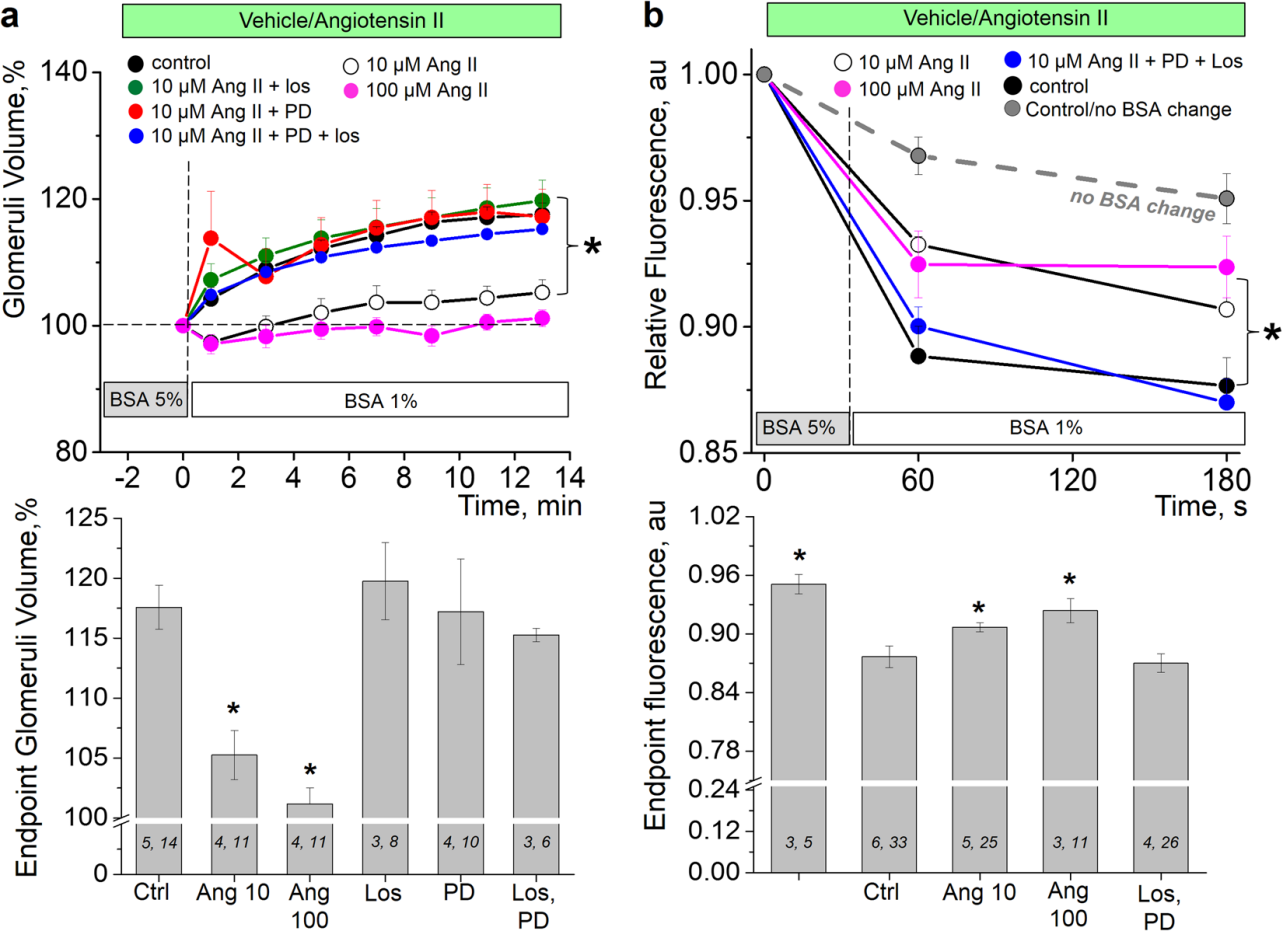
Effects of ATR blockers on rat glomeruli permeability and volume changes. (**a**) Activation of AT receptors by 10 or 100 µM of Ang II significantly suppresses glomeruli ability to respond to oncotic gradient changes evoked by solution change from 5% to 1% BSA (black, white and magenta data points represent control (vehicle), and 10 or 100 µM of Ang II, respectively). Inhibition of either AT_1_ or AT_2_ receptors with 10 µM losartan or 1 µM PD 123319, respectively (green and red data points) or both antagonists together (blue data points) restores normal glomeruli function and attenuates permeability. Summary graph for the endpoint glomeruli volume is shown on the lower panel. (**b**) Pre-application of Ang II significantly inhibits changes in glomerular FITC fluorescence and corresponding water flux across the capillary wall in response to changes in oncotic gradient. Application of ATRs blockers restores normal renal hemodynamics and attenuates glomerular permeability to protein. Summary graph for the endpoint glomeruli fluorescence is shown on the lower panel. (*i*,*j*) noted on graph is number of animals (*i*) and glomeruli (*j*) analyzed in each group, respectively. *P < 0.05 versus control experiment.

Further, in our experimental model we tested the complex effect of Ang II on the loss of intercapillary albumin across the capillary wall. As seen in Fig. 2a, solution change from 5% to 1% BSA results in an average increase of glomerular volume by 15%. However, this effect is significantly attenuated or completely precluded when glomeruli are pre-incubated with 10 or 100 μM Ang II for 30 min. Furthermore, glomerular volume change was restored by treatment with AT_1_R or AT_2_R inhibitors (10 µM losartan and 1 µM PD123319, respectively) either applied separately or together. In our experiments Ang II (in the presence or absence of ATR inhibitors) was added to the solution before the changes of BSA solutions (and was also kept in the media during oncotic gradient change) since Ang II can cause glomerular contraction.

Additionally, changes in glomerular FITC fluorescence (reflecting water flux across the capillary wall in response to changes in oncotic gradient) was inhibited by pre-incubation with Ang II (Fig. 2b). The application of ATR blockers restored this effect and attenuated glomerular permeability to protein. In order to assess the contribution of photobleaching into the observed changes, TRITC fluorescence was recorded continuously and compared to experimental recordings. As seen from Fig. 2b (grey dotted line), photobleaching was not significant and minimally affects the obtained data.

Therefore, both changes in glomerular volume (Fig. 2a) and FITC fluorescence (Fig. 2b) suggest that Ang II, working via both ATRs prevents glomerular dynamics in response to an oncotic gradient generated by BSA, and directly modulates glomerular permeability to albumin through the described mechanism. Thus, from these experiments we can conclude that both sides of this process–vasculature and podocytes–are involved and affected by activation of AT receptors.

### TRPC6 calcium channels are involved in glomeruli volume changes evoked by Ang II

**Figure 3:**
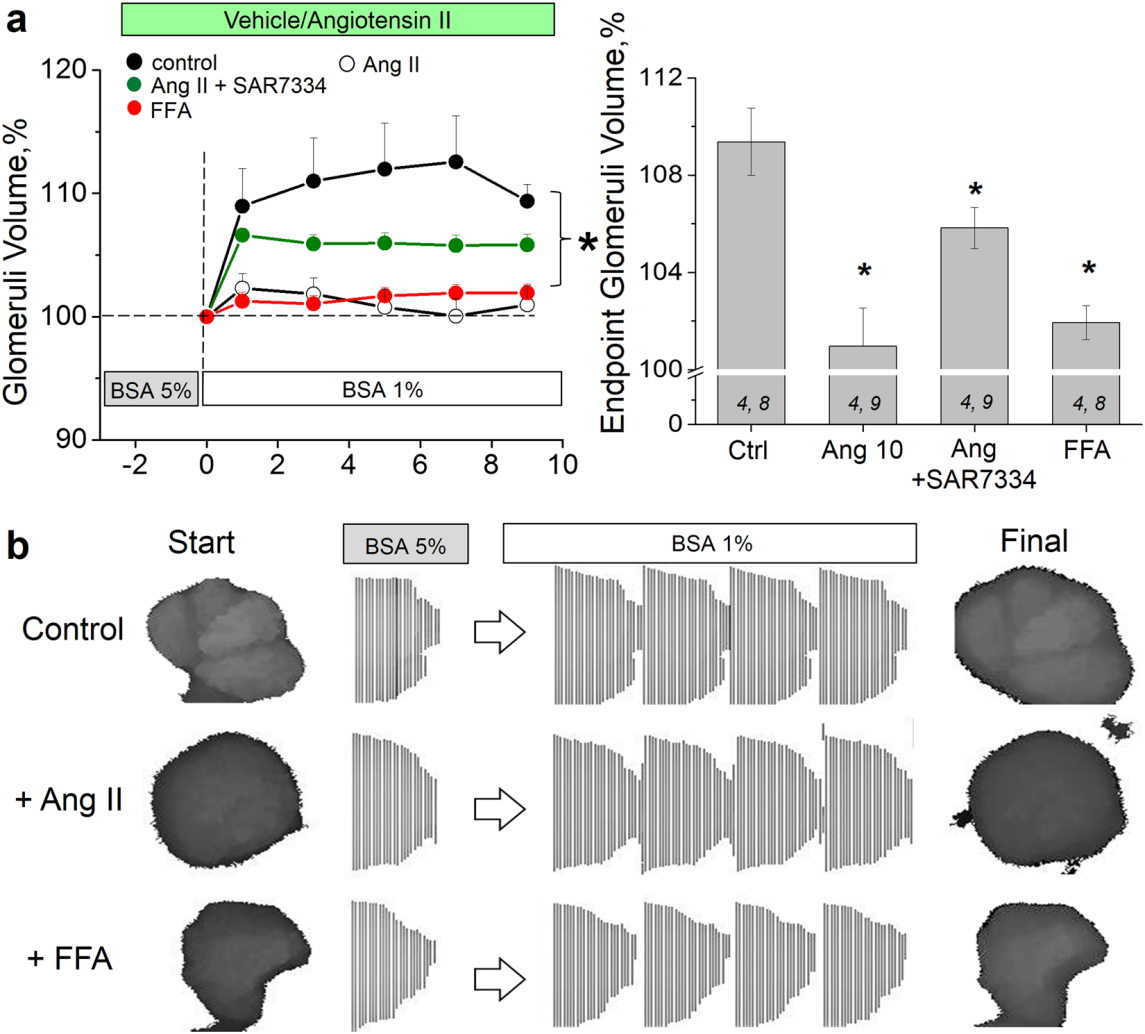
Effects of flufenamic acid and SAR7334 on rat glomeruli volume. (**a**) The effects of activation of TRPC6 channels by flufenamic acid (100 µM, FFA) or their blockage by SAR7334 (1 µM) on Ang II–induced glomeruli volume dynamics (left panel), and a summary plot for the endpoint glomeruli volume (right panel). (**b**) Representative reconstituted glomerular volumes and combined z-stacks illustrating the changes in the volume of a single glomerulus during the solution change from 5% to 1% BSA in control, and upon incubation with Ang II and FFA.

We demonstrated that activation of TRPC6 channels with flufenamic acid (a specific activator of TRPC6^39^) affects glomerular volume dynamics in a manner similar to that seen upon stimulation of glomeruli with Ang II (see Fig. 3a). Figure 3b illustrates the changes in glomerular volume in control and upon stimulation of TRPC6 channels by Ang II or FFA (reconstituted from the z-stacks acquired over time). Additionally, we tested the effect of SAR7334 (a novel specific TRPC6 channel inhibitor^40, 41^) on glomerular volume dynamics, and report here (see Fig. 3a) that blocking TRPC6 is able to partially restore glomerular volume change seen in the absence of Ang II. Therefore, these data allow to conclude that TRPC6 channels are, at least in part, involved in regulation of glomerular volume dynamics.

### Ang II–dependent calcium flux in the rat podocytes is mediated via calcium channels

**Figure 4:**
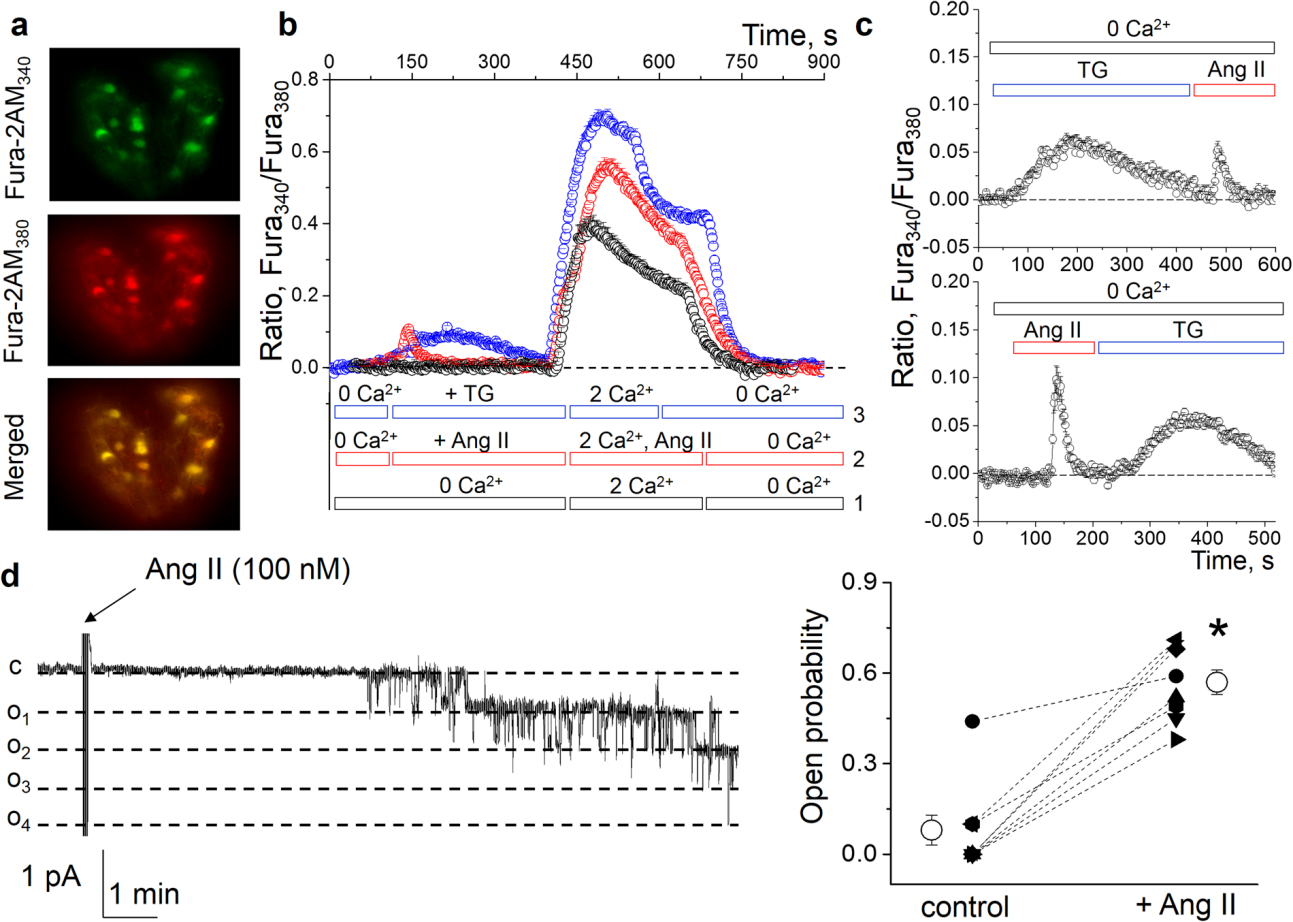
Ang II evokes [Ca^2+^]_i_ elevation and activates TRPC channels in the podocytes of the freshly isolated rat glomeruli. (**a**) Images of the Fura-2AM-loaded glomeruli obtained by fluorescence microscopy (examples of the fluorescence signals at 340 and 380 nm, and an image merged with brightfield view are shown). (**b**) Representative time course of the changes in [Ca^2+^]_i_ determined in podocytes of the freshly isolated glomeruli by the ratio of Fura_340_/Fura_380_ fluorescence, under three different conditions: (1) modulations in [Ca^2+^]_i_ induced by changes of medium containing 0 to medium with 2 mM Ca^2+^ (black values); (2) medium containing 0 or 2 mM Ca^2^ supplemented with 1 μM Ang II (red values); and (3) medium containing 10 μM thapsigargin (TG, as shown; blue values). Please note a transient in response to Ang II in Ca^2+^-free media (associated with store depletion). N = 4 animals per group. (**c**) Ang II-stimulated [Ca^2+^]_i_ peak is inhibited after calcium store depletion with TG. Shown is a [Ca^2+^]_i_ response after depletion of the intracellular store with TG and consecutive treatment with Ang II, and a reverse experiment. (**d**) Representative continuous current trace from a cell-attached patch containing endogenous TRPC channels. Arrow demonstrates addition of Ang II (100 nM) to the external bath solution. The patch was held at a −60 mV test potential during the course of experiment. The c and o_i_ denote closed and open current levels, respectively. Right panel shows a summary graph for the channels’ open probability (*P*_*o*_) before and after application of Ang II. *P < *0.05* versus before Ang II.

Here we monitored intracellular calcium changes ([Ca^2+^]_i_) in response to Ang II in isolated Wistar rat glomeruli loaded with Fura-2AM (Fig. 4a). Figure 4b demonstrates the effects of solution change from calcium-free to the solution containing 2 mM calcium, and back (black trace). Application of Ang II (1 μM, Fig. 4b, red trace) in the calcium-free solution evoked a transient that was observed for up to a 2 min period and should be attributed to intracellular store depletion. Further solution change to 2 mM CaCl_2_ caused a significantly more enhanced calcium influx compared to Ang II–untreated glomeruli (black trace). Application of 10 μM thapsigargin (TG) that causes intracellular calcium store depletion resulted in a sustainable ratio elevation in the calcium-free solution followed by a calcium influx in presence of 2 mM CaCl_2_ (Fig. 4b, blue trace).

In order to define a source responsible for the Ang II-induced [Ca^2+^]_i_ flux, we also used TG to selectively inhibit the endoplasmic reticulum Ca^2+^-ATPase and cause depletion of TG-sensitive Ca^2+^ stores. Cells were first treated with TG to deplete stores and the effects of application of Ang II on [Ca^2+^]_i_ was evaluated. We found that Ang II induced an increase in [Ca^2+^]_i_ after depletion of the intracellular stores (Fig. 4c), and vice versa, however if store depletion with TG occurred first, the effect of Ang II on [Ca^2+^]_i_ was substantially lower. Hence, Ang II-mediated [Ca^2+^]_i_ increase in podocytes is mediated both via influx through the membrane-residing ion channels, and calcium store depletion.

We have shown previously that Ang II activates native TRPC6 channels in mouse podocytes^31^ and type 1 diabetic STZ-SS rats^21^. Here we confirmed this effect and assessed TRPC activity in the podocytes of the normal Sprague-Dawley rat glomeruli with patch clamp electrophysiology. Figure 4d illustrates a representative time course of TRPC channel current activity and summary graph of changes in the channel open probability in the isolated glomerulus following addition of Ang II (100 nM). Application of Ang II resulted in an acute increase in the channel open probability (*P*_*o*_) in this native preparation.

### Ang II in the podocytes works via both AT_1_ and AT_2_ receptors

**Figure 5:**
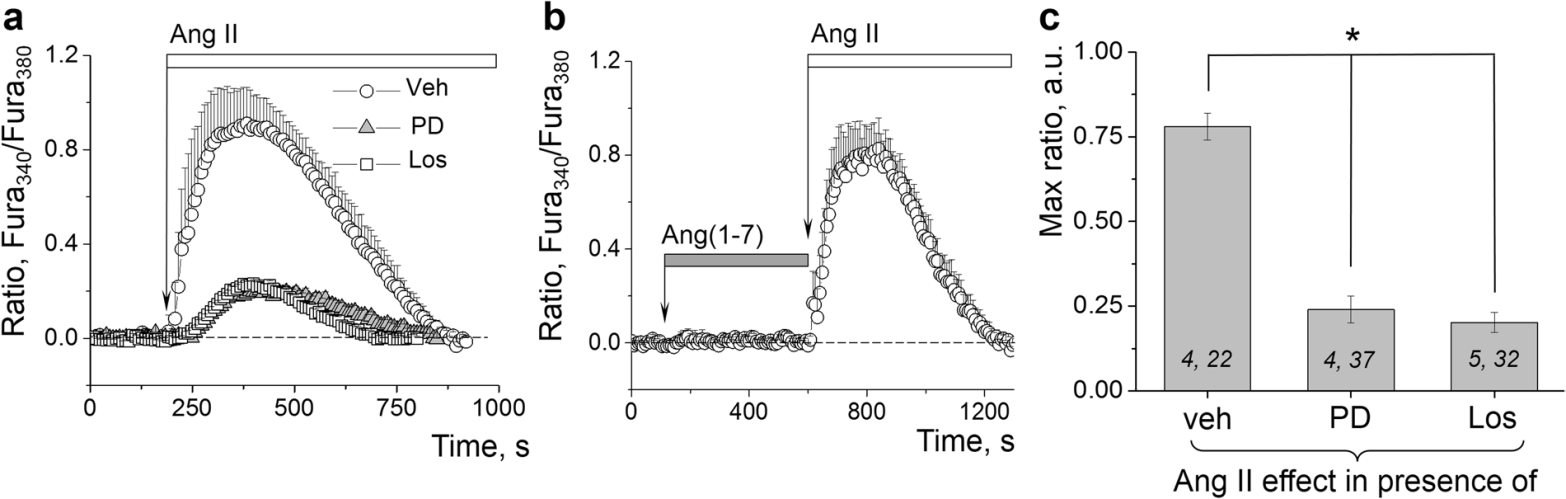
Ang II–dependent calcium influx in the podocytes is precluded by ATR inhibition. (**a**) Representative effects of losartan (Los, 10 µM, 30 min incubation) or PD 123319 (PD, 1 µM, 10 min), on Ca^2+^ transients evoked in the podocytes by 10 µM Ang II. (**b**) Illustrates the lack of an acute effect of a Mas receptor agonist Ang(1–7) on calcium influx in podocytes (concentration applied was 10 µM). (**c**) Summarized data for the effects of Ang II on calcium influx in the podocytes in the absence or presence of losartan or PD. (*i*,*j*) noted on graph is number of animals (*i*) and podocytes (*j*) analyzed in each group. *P < 0.01 versus Ang II.

Figure 5a and c demonstrate inhibition of the calcium influx in response to Ang II when glomeruli are pretreated with AT_1_R and AT_2_R blockers losartan (10 µM, 30 min) and PD 123319 (1 µM, 10 min), respectively. These results demonstrate that both AT_1_R and AT_2_R are mediating the action of Ang II in the podocytes. Additionally, we tested the involvement of the Mas receptor (a receptor for Ang-(1–7), a biologically active product of the RAS cascade) into the regulation of calcium handing in the podocytes. Acute application of up to 50 µM of Ang-(1–7) in the calcium-containing solution did not evoke any calcium transients in the podocytes (Fig. 5b).

### TRPC6 channels are responsible for Ang II–evoked calcium influx in rat podocytes

**Figure 6:**
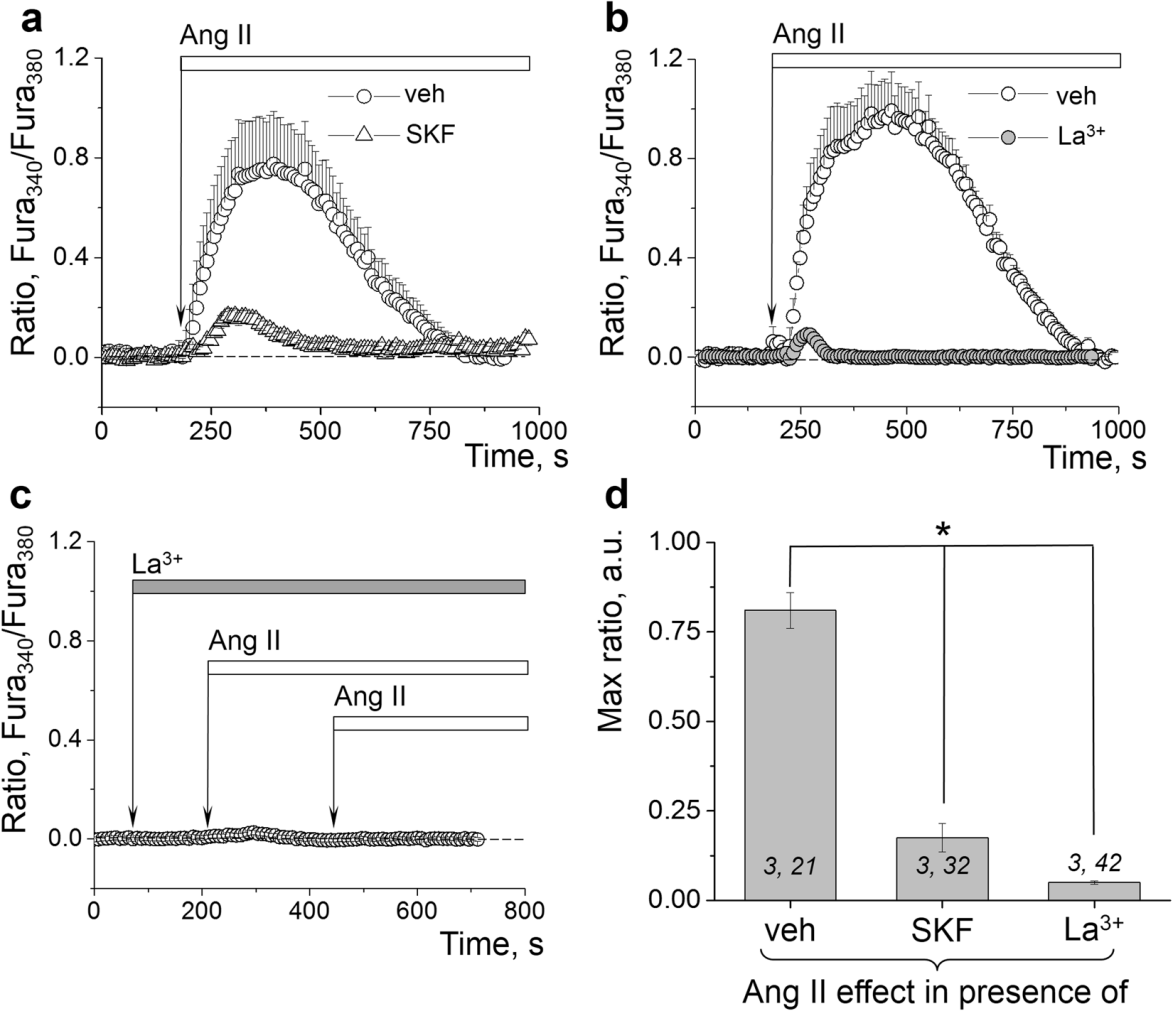
Ang II–dependent calcium influx in the podocytes mediated via TRPC channels. (**a**) Representative effects of the TRPC channel blocker SKF 96365 (SKF, 1 µM, 5 min) on Ca^2+^ transients evoked in the podocytes by 10 µM Ang II. (**b**) Representative transient demonstrating that 50 µM LaCl_3_ (upon 10 min pre-incubation) precludes the increase in calcium influx in response to Ang II in the podocytes. (**c**) Transient illustrating the lack of a potentiating effect of LaCl_3_ on calcium influx, and immediate inhibitory effect on Ang II-evoked calcium transients (10 µM Ang II was applied twice consecutively). (**d**) Graph summarizing the effects of Ang II on calcium influx in the podocytes in presence of SKF or LaCl_3_. (*i*,*j*) noted on graph is number of animals (*i*) and podocytes (*j*) analyzed in each group. *P < 0.01 versus Ang II.

We tested which TRPC channels are responsible for the major extracellular calcium influx in the rat podocytes. As seen from Fig. 6a, a pan-TRPC channels’ inhibitor SKF96365 (1 µM) largely prevented calcium influx in response to Ang II. We further tested the effects of La^3+^ on Ang II–mediated calcium influx. La^3+^ is known to have differential effects on members of the TRPC family: it can potentiate currents through TRPC4 and TRPC5 channels, and inhibit other TRPC family members. As seen from Fig. 6b and c, pre-incubation with 50 μM LaCl_3_ (10 min) precluded the calcium transient in response to Ang II; acute application of LaCl_3_ also failed to potentiate calcium influx in the podocytes. Therefore, these data exclude TRPC4 and TRPC5 channels as potential targets of Ang II.

**Figure 7:**
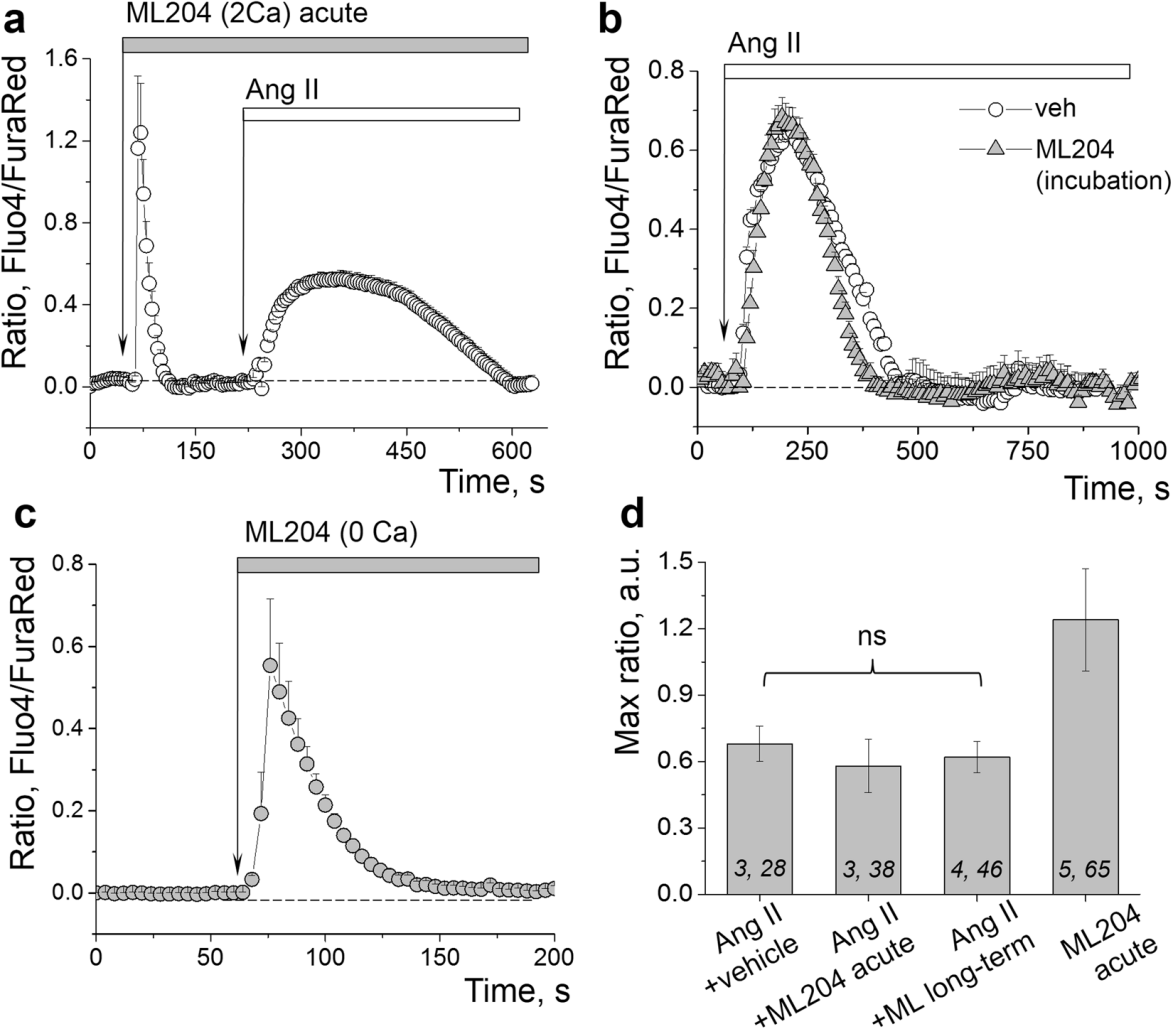
The effects of ML204 on calcium influx in the podocytes. (**a**) The representative effect of acute applications of 20 µM ML204 followed by 10 µM Ang II on Ca^2+^ level in the podocytes (in calcium-containing media). (**b**) Representative effect of ML204 (20 µM, 10 min incubation) on Ca^2+^ transient evoked in the podocytes by 10 µM Ang II. (**c**) The representative effect of the acute application of 20 µM ML204 on Ca^2+^ level in the podocytes in calcium-free solution. (**d**) Graph summarizing the effects of Ang II on calcium influx in the podocytes in presence of ML204, or an acute application of ML204 alone. (*i*,*j*) noted on graph is number of animals (*i*) and podocytes (*j*) analyzed in each group. *ns* denotes no statistically significant difference versus Ang II application in the absence of the inhibitor.

**Figure 8:**
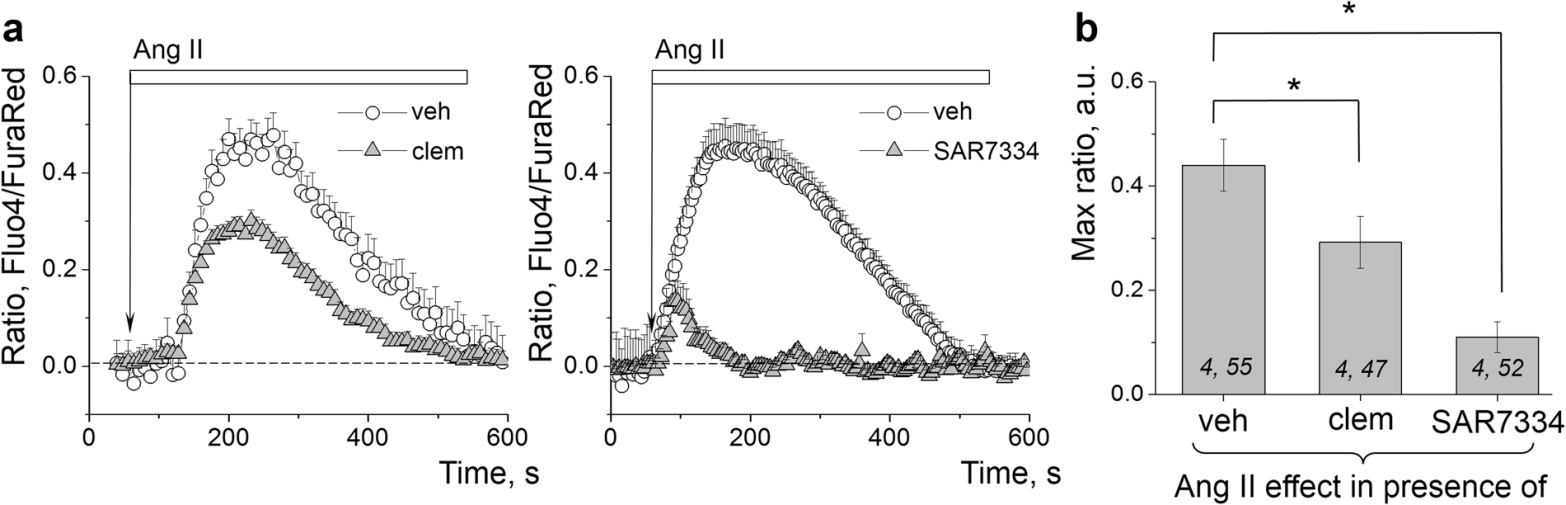
The involvement of TRPC6 channels into the Ang II–dependent calcium influx in the podocytes. (**a**) Representative effects of the TRPC channel blockers clemizole hydrochloride (Clem, 5 µM, 10 min) and SAR7334 (1 µM, 10 min) on Ca^2+^ transients evoked in the podocytes with 10 µM Ang II. (**b**) Graph summarizing the effects of Ang II on calcium influx in the podocytes in presence of clemizole and SAR7334. (*i*,*j*) noted on graph is number of animals (*i*) and podocytes (*j*) analyzed in each group. *P < 0.05 versus Ang II.

Additional experiments were aimed at further delineating whether TRPC5 is involved in the Ang II–evoked calcium transient in the isolated glomeruli. To test this, we employed pre-incubation or acute application of ML204 (20 µM), a known blocker of TRPC4 channels (IC_50_ values for TRPC4 are 0.96 and 2.6 μM in fluorescent and electrophysiological assays, respectively), which exhibits 19-fold selectivity against TRPC6 and 9-fold selectivity against TRPC5. Figure 7 demonstrates that upon acute application of ML204 in calcium-containing solution the response to a following application of Ang II is not affected (Fig. 7a); furthermore, Ang II–induced calcium response is preserved when glomeruli are pre-incubated with ML204 for 20 min (Fig. 7b). Additionally, it should be noted that ML204 produced an acute and potent release of calcium from the intracellular stores (Fig. 7c illustrates a response to an acute application of ML204 in the calcium-free solution), and therefore is unlikely to be a specific TRPC5 channel inhibitor. We next tested other TRPC channels inhibitors, clemizole hydrochloride (5 µM, known IC_50_ values are 1.1, 6.4, 9.1, 11.3 and 26.5 μM for TRPC5, TRPC4, TRPC3, TRPC6 and TRPC7 respectively) and a specific TRPC6 inhibitor SAR7334 (used 1 μM; is known to inhibit TRPC6, TRPC3 and TRPC7-mediated Ca^2+^ influx into cells with IC_50_ s of 9.5, 282 and 226 nM, whereas TRPC4 and TRPC5-mediated Ca^2+^ entry is not affected^40^). As reported on Fig. 8, clemizole hydrochloride inhibited approximately 30% of the calcium transient evoked by Ang II, whereas SAR7334 resulted in a major block of the Ang II–evoked calcium influx.

## Discussion

Ang II is among the recognized circulating factors that can be detrimental to podocytes and induce damage to the GFB^23, 27, 42, 43^. Recent data obtained using sophisticated *in vivo* techniques showed that there is a cell-to-cell calcium signaling that travels as a calcium wave to all cells of the glomerulus, including the most distant podocytes^36, 44^. The oscillatory glomerular contractions observed in living freshly isolated glomeruli were hypothesized to reflect interplay among different cells of the glomerulus, including podocytes^45^. Current study was specifically designed to characterize the effects of Ang II on a glomerulus at different regulatory levels–individual ion channels in the membrane, calcium handling within the glomerular cells, and the dynamics of the whole glomerulus.

We employed a thorough and comprehensive method to assess the functionality of GFB integrity by using fluorescent dextrans, which recently became popular for *in vivo* and *ex vivo* applications^45–47^. Here we modified the classical technique developed by Savin *et al*.^38^, and an employed combination of fast confocal scanning and two fluorescent dextrans allowed us to clearly show changes in volume of isolated glomeruli that occur as a result of the change in osmotic gradient (see Figs 1 and 2). Although the method of albumin reflection coefficient measurement using isolated rat glomeruli was published in 1992 and has been since widely used by many researchers^48–51^, the calculations were based on the measurements of the diameter of the isolated glomerulus (which is not a sphere, but rather an ellipsoid with complex geometry), and most publications lacked representative images that would illustrate the actual changes. A recent report using a similar fluorescent dilution technique^45^ reported an up to 68% change in the fluorescence intensity of the glomeruli in response to oncotic gradient. The dilution of fluorescent particles by water entrance in glomeruli is proportional to the volume changes, thus, such an increase should promote a similar glomeruli volume increase. In contrast, our approach shows direct correlation between florescence intensity and volume increase of the glomerulus, furthermore, the observed change was less than 20%, and can be precisely calculated independently from the geometrical form of the glomerulus.

The preparation of the freshly isolated rat glomeruli used here has advantages compared to cultured cells, and, as accurately stated by Savin *et al*., allows assessing the direct effects of the studied drugs “*independent of systemic or intrarenal hemodynamic effects*, *of secondary effects due to altered intracapillary pressures or perfusion or filtration rates*, *and of intrarenal feedback from extraglomerular vasculature or tubules*”^38^. This unique preparation enabled us to reveal that on the one hand, Ang II via both AT_1_ and AT_2_ receptors affects intracellular calcium handling within glomerular epithelial cells, which might result in podocytopenia and subsequent proteinuria. On the other hand, we have shown that Ang II mediates the contractile properties of the glomeruli, which reflects the ability of the glomerulus to filter plasma and retain protein in the bloodstream.

Previous studies demonstrated that administration of the AT_2_ receptor antagonist PD123319 is associated with beneficial effects on a range of renal functional and structural parameters^52^. Moreover, the combination of both AT_1_ and AT_2_ receptor antagonists may provide additional renal protection than either antagonist alone. In contrast, other groups report that overexpression of AT_2_ led to reduced glomerular injuries in mice with 5/6 nephrectomy (without influence on blood pressure)^53^. A recent multi-photon study^54^ demonstrated by measuring albumin glomerular sieving coefficient (GSC) that the AT_2_ receptor can partially attenuate the proteinuric effects of the AT_1_ receptor, and, therefore, AT_1_ and AT_2_ receptors may exert opposing effects on the GSC. In our experiments, however, inhibition of the AT_1_ or AT_2_ Ang II receptor subtypes by losartan and PD123319, respectively, exhibited the same effects. Furthermore, blocking both AT_1_ and AT_2_ together did not result in further attenuation of the glomerulus contraction dynamics. These observations are intriguing and require further studies, especially in the animals genetically deficient for the either subtype of the Ang II receptor. The possible reported antagonism between AT_1_ and AT_2_ receptor^55^ might play a role in the mechanism that would counteract the onset of proteinuric kidney diseases. Furthermore, recent reports provide evidence that the commonly accepted scheme of physiological vasorelaxation balance between a constricting effect of AT_1_ and a dilating effect of AT_2_ is too simplistic and does not always reflect experimental evidence^56^. Further investigation with more selective and specific AT_2_ receptor pharmacology is needed to unveil the molecular mechanisms of AT_1_–AT_2_ receptors dimerization which might affect the consecutive signal transduction^57^.

Activation or inhibition of the TRPC calcium channel with flufenamic acid and SAR7334, respectively, confirmed that Ang II–stimulated calcium flux via TRPC channels is at least in part mediating glomerular volume dynamics (Fig. 3). It is clear that volume regulation of glomeruli is not only dependent on podocytes but on many cell types (e.g. mesangial cells); further experiments of this study were devoted to an attempt to differentiate between TRPC5 and/or TRPC6 channels input in the calcium influx evoked by Ang II specifically in the podocytes. It has been debated whether TRPC5 or TRPC6 channels are responsible for conducting the calcium signal in the podocyte in pathological states; a recent review by Wieder and Greka^29^ suggested that TRPC6 channels have homeostatic function maintaining GFB integrity, whereas TRPC5 is induced in a disease state. Our previous studies using *TRPC6* knockout mice^31^ demonstrated that TRPC6 is essential for the Ang II–mediated calcium transient in the podocytes, at least in the normal conditions. Here we employed a variety of pharmacology, including well-known compounds SKF 96365 (pan-TRPC channel blocker), La^3+^ (known to potentiate TRPC4 and TRPC5 and inhibit other TRPC family members) as well as clemizole hydrochloride, a novel product that exerts 10-fold specificity towards TRPC5 over TRPC6^58^, and a small molecule inhibitor ML204 previously used to support the role of TRPC5 in the podocyte damage^59^ (reported to be highly selective for TRPC5 at 10 µM, used in concentration of 10 to 30 µM). Our data revealed that neither acute application nor a 15 min pre-treatment with ML204 at 20 µM modulate Ang II–mediated calcium transients in the podocytes of the freshly isolated glomeruli. Clemizole hydrochloride used in a concentration of 5 µM (IC_50_ values are 1.1 and 11.3 μM for TRPC5 and TRPC6, respectively) attenuated the response to Ang II by approximately 30%. This concentration exceeds maximum dose required to completely block TRPC5 and sufficient to partially block TRPC6 channels. La^3+^ also inhibited, but did not potentiate Ang II-induced current. Nevertheless, there is not much evidence to bolster the conclusion that TRPC5 channels are significantly involved in the transduction of the calcium signal in the podocytes. Furthermore, a novel specific TRPC6 inhibitor, SAR7334, which does not affect TRPC5, resulted in a potent block of Ang II–evoked calcium transient in the podocytes. Taking into consideration that there is a human gain-of-function mutation in the TRPC6 channel which leads to FSGS^2^, it is highly likely that this channel is also implicated in the disease-associated events; however, the hypothesis that TRPC5 channel can be induced in the disease state is intriguing and still requires further investigation which is beyond the scope of the current study. Of note, the described glomerular volume measurement technique with some dexterity can be used on mouse glomeruli. Therefore, more precise characterization of the involvement of TRCP6 channels can be performed using mouse knockout models, which are more readily available than genetically modified rats.

In summary, GFB should be regarded as a single entity consisting of different types of cells that depend on each other; its integrity can be damaged when just one cell type is affected, and then deteriorated as a result of the broken intracellular signaling connections. The functional properties of GFB should be further comprehensively studied using contemporary techniques, such as multi-photon microscopy, that allows investigators the opportunity to study dynamic changes in the glomerulus and within the functioning living kidney.

## Methods

### Glomeruli volume dynamics assay

All methods were carried out in accordance with the approved guidelines. For the measurements of the glomeruli volume changes rats were infused with FITC-dextran (150 kDa dissolved in 0.9% NaCL, 50 mg/mL, TdB Consultancy AB, Uppsala, Sweden) via the femoral vein. Then, rat kidneys were harvested; glomeruli were isolated by differential sieving and stored on ice in a 5% BSA/RPMI solution with glomeruli non-permeable 150 kDa TRITC labeled dextran. Then, glomeruli were attached to poly-L-lysine covered glass coverslips for further confocal microscopy imaging. Fluorescence intensities detected with the FITC and TRITC filters were monitored by confocal laser scanning microscope system Nikon A1-R, and represented inner and outer glomeruli space, respectively. A Z-stack of 26 consecutive focal planes (73.83 µm) was collected every 2 minutes, which allowed reconstructing fluorescence within glomerular capillaries (FITC), and glomeruli volume (TRITC) using Fiji image processing package (ImageJ 1.47 v, National Institute of Health, USA) and Origin Pro 9.0 (OriginLab, Northampton, MA). Water dilution of inner glomeruli fluorescence and volume changes created by the oncotic pressure induced by switching the surrounding medium from 5% into 1% BSA was monitored by 3D imaging throughout the experiment. For glomeruli volume reconstitution, TRITC signal was inverted, and each focal plane was processed by the Analyze Particles module (ImageJ). Finally, glomeruli volume was calculated by the integration of the obtained focal planes using OriginPro software. Total glomeruli FITC fluorescence within a z-stack was calculated as a sum of slices’ intensities.

### Isolation of the rat glomeruli, electrophysiology and intracellular calcium imaging

Experimental procedures were performed as described in our earlier publications using confocal or epifluorescence imaging and single-channel patch-clamp electrophysiology^21, 31, 60^. Briefly, kidneys of 8 to 10-weeks-old male Wistar or Sprague Dawley rats were cleared from the blood, removed and decapsulated; the cortex was isolated, minced and sequentially pushed through steel sieves using the culture medium solution RPMI1640 (Invitrogen, Inc) with 5% BSA. After isolation glomeruli were used either for single-channel patch-clamping and/or microscopy experiments. For electrophysiology cover glasses with attached glomeruli were placed into a perfusion chamber and mounted on an inverted Nikon Ti-S microscope. After a high resistance seal was obtained, cell-attached recording was performed immediately in the solutions described previously^21, 31^. Single-channel unitary current (i) was determined from the best-fit Gaussian distribution of amplitude histograms. Activity was analyzed as NP_o_ = I/i, where I is the mean total current in a patch and i is unitary current at this voltage. P_o_ (open probability) was calculated by normalizing NP_o_ for the total number of estimated channels (N) in the patch. The activity of the channels was first monitored in response to the voltage steps of 10 or 20 mV in the range of −90 mV to +60 mV in order to estimate the channel's conductance and I-V relationship. After that, the voltage was clamped at −60 mV and the channels’ activity was recorded for several minutes before application of Ang II. Animal use and welfare adhered to the NIH *Guide for the Care and Use of Laboratory Animals* following a protocol reviewed and approved by the IACUC at the Medical College of Wisconsin.

### Statistical analysis

Data are presented as mean ± s.e.m. The values of intracellular calcium ion concentration at every moment of time for individual cells were averaged by the number of regions registered in the experiment (*n* = 15–20). Data are compared using the Student’s (two-tailed) *t*-test, and *P* < 0.05 is considered significant.

## References

[1] Scott RP, Quaggin SE (2015). The cell biology of renal filtration. J. Cell Biol..

[2] Winn MP (2005). A mutation in the TRPC6 cation channel causes familial focal segmental glomerulosclerosis. Science.

[3] Winn MP, Daskalakis N, Spurney RF, Middleton JP (2006). Unexpected role of TRPC6 channel in familial nephrotic syndrome: does it have clinical implications?. J. Am. Soc. Nephrol..

[4] Krall P (2010). Podocyte-specific overexpression of wild type or mutant trpc6 in mice is sufficient to cause glomerular disease. PLoS ONE.

[5] Wang, L. *et al.* Gq signaling causes glomerular injury by activating TRPC6. *J. Clin. Invest.* **125**, 1913-26 (2015). 10.1172/JCI76767 PMC4463190 25844902

[6] Abkhezr M, Dryer SE (2014). Angiotensin II and canonical transient receptor potential-6 activation stimulate release of a signal transducer and activator of transcription 3-activating factor from mouse podocytes. Mol. Pharmacol..

[7] Anderson M, Roshanravan H, Khine J, Dryer SE (2014). Angiotensin II activation of TRPC6 channels in rat podocytes requires generation of reactive oxygen species. J. Cell. Physiol..

[8] Dryer SE, Reiser J (2010). TRPC6 channels and their binding partners in podocytes: role in glomerular filtration and pathophysiology. Am. J. Physiol. Renal Physiol..

[9] Roshanravan H, Dryer SE (2014). ATP acting through P2Y receptors causes activation of podocyte TRPC6 channels: role of podocin and reactive oxygen species. Am. J. Physiol. Renal Physiol..

[10] Greka A, Mundel P (2011). Balancing Calcium Signals through TRPC5 and TRPC6 in Podocytes. J. Am. Soc. Nephrol..

[11] Bakris GL, Kuritzky L (2009). Monitoring and managing urinary albumin excretion: practical advice for primary care clinicians. Postgrad. Med..

[12] Cowley AW (2013). Progression of glomerular filtration rate reduction determined in conscious Dahl salt-sensitive hypertensive rats. Hypertension.

[13] Molitoris BA (2010). Yet another advance in understanding albuminuria?. J. Am. Soc. Nephrol..

[14] Brinkkoetter PT, Ising C, Benzing T (2013). The role of the podocyte in albumin filtration. Nat. Rev. Nephrol.

[15] Kalluri R (2006). Proteinuria with and without renal glomerular podocyte effacement. J. Am. Soc. Nephrol..

[16] Pavenstadt H, Bek M (2002). Podocyte electrophysiology, *in vivo* and *in vitro*. Microsc. Res. Tech..

[17] Patrakka J, Tryggvason K (2009). New insights into the role of podocytes in proteinuria. Nat. Rev. Nephrol.

[18] Evans JF, Lee JH, Ragolia L (2009). Ang-II-induced Ca(2+) influx is mediated by the 1/4/5 subgroup of the transient receptor potential proteins in cultured aortic smooth muscle cells from diabetic Goto-Kakizaki rats. Mol. Cell. Endocrinol..

[19] Sonneveld R (2014). Glucose specifically regulates TRPC6 expression in the podocyte in an AngII-dependent manner. Am. J. Pathol..

[20] Ilatovskaya DV, Staruschenko A (2015). TRPC6 channel as an emerging determinant of the podocyte injury susceptibility in kidney diseases. Am. J. Physiol. Renal Physiol..

[21] Ilatovskaya DV (2015). Podocyte injury in diabetic nephropathy: implications of angiotensin II - dependent activation of TRPC channels. Sci Rep.

[22] Kobori H, Nishiyama A, Abe Y, Navar LG (2003). Enhancement of intrarenal angiotensinogen in Dahl salt-sensitive rats on high salt diet. Hypertension.

[23] Jefferson JA, Shankland SJ (2014). The pathogenesis of focal segmental glomerulosclerosis. Adv. Chronic Kidney Dis..

[24] Chi, X. *et al.* Losartan treating podocyte injury induced by Ang II via downregulation of TRPC6 in podocytes. *J. Renin Angiotensin Aldosterone Syst* (2015). 10.1177/1470320315573682 25795457

[25] Tian ML, Shen Y, Sun ZL, Zha Y (2015). Efficacy and safety of combining pentoxifylline with angiotensin-converting enzyme inhibitor or angiotensin II receptor blocker in diabetic nephropathy: a meta-analysis. Int. Urol. Nephrol..

[26] Eckel J (2011). TRPC6 enhances angiotensin II-induced albuminuria. J. Am. Soc. Nephrol..

[27] Nijenhuis T (2011). Angiotensin II contributes to podocyte injury by increasing TRPC6 expression via an NFAT-mediated positive feedback signaling pathway. Am. J. Pathol..

[28] Zhang H, Ding J, Fan Q, Liu S (2009). TRPC6 up-regulation in Ang II-induced podocyte apoptosis might result from ERK activation and NF-kappaB translocation. Exp. Biol. Med..

[29] Wieder, N. & Greka, A. Calcium, TRPC channels, and regulation of the actin cytoskeleton in podocytes: towards a future of targeted therapies. *Pediatr. Nephrol.* **31**, 1047–54 (2015). 10.1007/s00467-015-3224-1 PMC4840088 26490951

[30] Szabo T, Ambrus L, Zakany N, Balla G, Biro T (2015). Regulation of TRPC6 ion channels in podocytes - Implications for focal segmental glomerulosclerosis and acquired forms of proteinuric diseases. Acta Physiol. Hung..

[31] Ilatovskaya DV (2014). Angiotensin II has acute effects on TRPC6 channels in podocytes of freshly isolated glomeruli. Kidney Int..

[32] Fujiwara Y, Kikkawa R, Kitamura E, Takama T, Shigeta Y (1984). Angiotensin II effects upon glomerular intracapillary volume in the rat. Ren. Physiol.

[33] Savin VJ (1986). *In vitro* effects of angiotensin II on glomerular function. Am. J. Physiol..

[34] Sharma R, Sharma M, Vamos S, Savin VJ, Wiegmann TB (2001). Both subtype 1 and 2 receptors of angiotensin II participate in regulation of intracellular calcium in glomerular epithelial cells. J. Lab. Clin. Med..

[35] Sharma M, Sharma R, Greene AS, McCarthy ET, Savin VJ (1998). Documentation of angiotensin II receptors in glomerular epithelial cells. Am. J. Physiol..

[36] Peti-Peterdi J, Sipos A (2010). A high-powered view of the filtration barrier. J. Am. Soc. Nephrol..

[37] Hohne M (2013). Light microscopic visualization of podocyte ultrastructure demonstrates oscillating glomerular contractions. Am. J. Pathol..

[38] Savin VJ, Sharma R, Lovell HB, Welling DJ (1992). Measurement of albumin reflection coefficient with isolated rat glomeruli. J. Am. Soc. Nephrol..

[39] Jung S, Strotmann R, Schultz G, Plant TD (2002). TRPC6 is a candidate channel involved in receptor-stimulated cation currents in A7r5 smooth muscle cells. Am. J. Physiol. Cell Physiol.

[40] Maier T (2015). Discovery and pharmacological characterization of a novel potent inhibitor of diacylglycerol-sensitive TRPC cation channels. Br. J. Pharmacol..

[41] Chauvet S (2016). Pharmacological Characterization of the Native Store-Operated Calcium Channels of Cortical Neurons from Embryonic Mouse Brain. Front Pharmacol.

[42] Arora P (2015). Renin-Angiotensin-Aldosterone System Blockers in Elderly Adults with Chronic Kidney Disease without Diabetes Mellitus or Proteinuria. J. Am. Geriatr. Soc..

[43] Hayashi K (2015). Renin-angiotensin blockade resets podocyte epigenome through Kruppel-like Factor 4 and attenuates proteinuria. Kidney Int..

[44] Peti-Peterdi J (2006). Calcium wave of tubuloglomerular feedback. Am. J. Physiol. Renal Physiol..

[45] Fan F (2015). Fluorescence dilution technique for measurement of albumin reflection coefficient in isolated glomeruli. Am. J. Physiol. Renal Physiol.

[46] Yeo NC (2015). Shroom3 contributes to the maintenance of the glomerular filtration barrier integrity. Genome Res..

[47] Sandoval, R. M. & Molitoris, B. A. Quantifying glomerular permeability of fluorescent macromolecules using 2-photon microscopy in Munich Wistar rats. *J. Vis. Exp.* e50052 (2013). 10.3791/50052 PMC3664960 23628966

[48] Sharma R (2007). Chymase increases glomerular albumin permeability via protease-activated receptor-2. Mol. Cell. Biochem..

[49] Williams JM, Sharma M, Anjaiahh S, Falck JR, Roman RJ (2007). Role of endogenous CYP450 metabolites of arachidonic acid in maintaining the glomerular protein permeability barrier. Am. J. Physiol. Renal Physiol.

[50] Dahly-Vernon AJ (2005). Transforming growth factor-beta, 20-HETE interaction, and glomerular injury in Dahl salt-sensitive rats. Hypertension.

[51] Saleh MA (2010). Endothelin-1 increases glomerular permeability and inflammation independent of blood pressure in the rat. Hypertension.

[52] Bonnet F (2002). Renal expression of angiotensin receptors in long-term diabetes and the effects of angiotensin type 1 receptor blockade. J. Hypertens..

[53] Hashimoto N (2004). Overexpression of angiotensin type 2 receptor ameliorates glomerular injury in a mouse remnant kidney model. Am J Physiol Renal Physiol.

[54] Schiessl IM, Castrop H (2013). Angiotensin II AT2 receptor activation attenuates AT1 receptor-induced increases in the glomerular filtration of albumin: a multiphoton microscopy study. Am J Physiol Renal Physiol.

[55] Suzuki K (2007). Angiotensin II type 1 and type 2 receptors play opposite roles in regulating the barrier function of kidney glomerular capillary wall. Am. J. Pathol..

[56] Verdonk K (2012). Compound 21 induces vasorelaxation via an endothelium- and angiotensin II type 2 receptor-independent mechanism. Hypertension.

[57] Henrion D (2012). Why do we need a selective angiotensin II type 2 receptor agonist?. Hypertension.

[58] Richter JM, Schaefer M, Hill K (2014). Clemizole hydrochloride is a novel and potent inhibitor of transient receptor potential channel TRPC5. Mol. Pharmacol..

[59] Schaldecker T (2013). Inhibition of the TRPC5 ion channel protects the kidney filter. J. Clin. Invest..

[60] Ilatovskaya, D. V., Palygin, O., Levchenko, V. & Staruschenko, A. Single-channel Analysis and Calcium Imaging in the Podocytes of the Freshly Isolated Glomeruli. *J. Vis. Exp.* e52850 (2015). 10.3791/52850 PMC4544950 26167808

